# Serum phosphate and magnesium in children recovering from severe acute undernutrition in Ethiopia: an observational study

**DOI:** 10.1186/s12887-016-0712-9

**Published:** 2016-11-05

**Authors:** Anne-Louise Hother, Tsinuel Girma, Maren J. H. Rytter, Alemseged Abdissa, Christian Ritz, Christian Mølgaard, Kim F. Michaelsen, André Briend, Henrik Friis, Pernille Kæstel

**Affiliations:** 1 Department of Nutrition, Exercise and Sports, University of Copenhagen, Rolighedsvej 30, Frederiksberg C, DK-1958 Denmark; 2 Department of Pediatric and Child Health, Jimma University Specialized Hospital, Jimma, Ethiopia; 3 Department of Laboratory Sciences and Pathology, Jimma University Specialized Hospital, Jimma, Ethiopia; 4 Department for International Health, University of Tampere, Tampere, Finland

**Keywords:** Refeeding syndrome, Hypophosphatemia, Hypomagnesemia, Therapeutic feeding, Nutritional rehabilitation, Severe acute malnutrition

## Abstract

**Background:**

Children with severe acute malnutrition (SAM) have increased requirements for phosphorus and magnesium during recovery. If requirements are not met, the children may develop refeeding hypophosphatemia and hypomagnesemia. However, little is known about the effect of current therapeutic diets (F-75 and F-100) on serum phosphate (S-phosphate) and magnesium (S-magnesium) in children with SAM.

**Methods:**

Prospective observational study, with measurements of S-phosphate and S-magnesium at admission, prior to rehabilitation phase and at discharge in children aged 6–59 months admitted with SAM to Jimma Hospital, Ethiopia. Due to shortage of F-75, 25 (35 %) children were stabilized with diluted F-100 (75 kcal/100 ml).

**Results:**

Of 72 children enrolled, the mean age was 32 ± 14 months, and edema was present in 50 (69 %). At admission, mean S-phosphate was 0.92 ± 0.34 mmol/L, which was low compared to normal values, but increased to 1.38 ± 0.28 mmol/L at discharge, after on average 16 days. Mean S-magnesium, at admission, was 0.95 ± 0.23 mmol/L, and increased to 1.13 ± 0.17 mmol/L at discharge. At discharge, 18 (51 %) children had S-phosphate below the normal range, and 3 (9 %) had S-phosphate above. Most children (83 %) had S-magnesium above normal range for children. Both S-phosphate and S-magnesium at admission were positively associated with serum albumin (S-albumin), but not with anthropometric characteristics or co-diagnoses. Using diluted F-100 for stabilization was not associated with lower S-phosphate or S-magnesium.

**Conclusion:**

Hypophosphatemia was common among children with SAM at admission, and still subnormal in about half of the children at discharge. This could be problematic for further recovery as phosphorus is needed for catch-up growth and local diets are likely to be low in bioavailable phosphorus. The high S-magnesium levels at discharge does not support that magnesium should be a limiting nutrient for growth in the F-100 diet. Although diluted F-100 (75 kcal/100 mL) is not designed for stabilizing children with SAM, it did not seem to cause lower S-phosphate than in children fed F-75.

## Background

Deficiencies of phosphorus, magnesium and other minerals are common in children with severe acute malnutrition (SAM) [1–5]. Refeeding with diets high in carbohydrate but with inadequate amount of phosphorus and magnesium can result in refeeding syndrome, characterized by hypophosphatemia and hypomagnesemia, sometimes resulting in respiratory or circulatory failure or even death [6, 7]. Although concentrations of serum phosphate (S-phosphate) and magnesium (S-magnesium) may not adequately reflect body status, low levels may still be suggestive of inadequate intake.

Treatment of children with SAM is divided in three phases; stabilization, transition and rehabilitation. The aim is first to stabilize the child and treat life-threatening complications, and then to feed the child intensively to allow for catch-up growth [8].

F-75 is formulated for the stabilization phase to be low in protein, fat and sodium. F-100 or ready-to-use therapeutic food is given during transition and rehabilitation phase and formulated to provide the nutrients needed during catch up growth. Many nutritional rehabilitation centers use commercial pre-mixed F-75 and F-100 products that simply need to be mixed with water, but F-75 and F-100 can also be made from locally available ingredients with an added vitamin-mineral mix (including potassium and magnesium but not phosphorus) [8].

When formulating these diets the phosphorus content was not considered. The phosphorus content thus varies from recipe to recipe depending on the ingredients used. The main source of phosphorus in these diets is the protein source; thus the amount of phosphorus depends on the amount and source of protein. The most widely used recipes are based on skimmed milk powder. The World Health Organization's (WHO) recipe for preparation of F-75 and F-100 from locally available ingredients (see [8] for more details) contain 2.5 g and 8.0 g of skimmed milk powder per 100 mL of F-75 and F-100, respectively. Assuming a phosphorus content of skimmed milk powder of 956 mg/100 g [9] a child fed 130 mL of locally prepared F-75 per kg per day during stabilization, gets 31 mg/kg/day of phosphorus and a child fed 200 mL/kg/day of F-100 during rehabilitation, gets 152 mg/kg/day of phosphorus.

Locally prepared F-75 thus provide limited amounts of phosphorous during stabilization, and substantially less than the 60 mg/kg/day recommended by WHO [8]. This combination of a high proportion of energy from carbohydrate and the low phosphorus content may be problematic [10]. Following new UN procurement specifications for F-75 [11], extrinsic phosphorus, in the form of a phosphorus salt has been added to the commercial pre-mixed F-75, to a level of 56 mg of phosphorus per 100 mL of F-75 (i.e. a child fed 130 mL of pre-mixed F-75 per kg per day, gets 73 mg/kg/day of phosphorus). The bioavailability of the phosphorus salt in children with SAM is unknown, but might be low due to decreased gastric acidity, and it is not clear if it is well absorbed [12–15]. Unlike phosphorus, magnesium is included in the standard mineral mix, used for local preparation of F-75, and both locally made and pre-mixed F-75 and F-100 are thus fortified with magnesium [16]. However, as the total body magnesium deficit might be greater than first anticipated, magnesium might still be limiting for growth in the F-100 diet [17].

In some situations, when F-75 is not available, F-100 is diluted to contain 75 kcal/100 ml, and used for stabilization. However, the composition of F-75 is quite different from diluted F-100 [18]. For instance, in F-75 the amount of energy derived from carbohydrate is much higher than in diluted F-100 (63 vs 41 E%). The total amount of phosphorus supplied by pre-mixed F-75 (complying with UN procurement specification, i.e. 56 mg of phosphorus per 100 mL) and diluted F-100, when feeding a volume of 130 ml/kg/day is 73 and 57 mg/kg/day, respectively.

To our knowledge, no other studies have evaluated the effect of F-75 and F-100, complying with UN specifications, on S-phosphate and S-magnesium concentrations during treatment of SAM.

We here present levels and predictors of S-phosphate and S-magnesium in Ethiopian children with SAM, treated according to current dietary regimen (i.e. including a stabilization, transition and rehabilitation phase) using F-75 and F-100 complying with UN specifications and in a group of children stabilized with diluted F-100 (75 kcal/100 mL) during a period of national shortage of F-75.

## Methods

### Study setting and participants

Children between 6 and 59 months of age admitted with nutritional edema and/or severe wasting (weight-for-height < 70 % of the median based upon the National Center for Health Statistics (NCHS) reference or Mid-upper-arm-circumference (MUAC) < 11.0 cm) were recruited for the study at the Nutritional Rehabilitation Unit (NRU) or critical ward of the pediatric unit of Jimma University Specialised Hospital (JUSH). Both children with complicated and uncomplicated SAM were treated at the NRU. Patients in shock, with severe respiratory difficulties or uncontrolled significant bleeding from any site were not included. If the parent or guardian gave consent, the child was examined and a blood sample was collected within the first 24 h after the child received the first feed.

### Anthropometric and clinical assessments

Anthropometric and clinical assessments were done, and blood samples were collected at admission, at start of rehabilitation phase and at discharge. Body weight was measured to the nearest 5 g using an electronic pediatric scale (Tanita BD 815 MA, Tokyo, Japan). Most children were too weak to stand at admission and stature of all children, regardless of age, was measured as recumbent length to the nearest 0.5 cm using a length board (SECA 416, Hamburg, Germany). Standing height was measured to the nearest cm for children longer than 100 cm (i.e. maximum length of the length board), using a stadiometer (SECA 214, Hamburg, Germany). Anthropometric z-scores based on the 2006 WHO child growth standard were calculated [19]. For calculation of weight-for-height and height-for-age z-scores in children of 24 months and older, height estimates were obtained by subtracting 0.7 cm from the recumbent length. Admission height or length was used for calculation of weight-for-height and height-for-age z-scores for all time points. MUAC was measured to the nearest 0.1 cm using an insertion tape (SECA 212, Hamburg, Germany). Presence of bilateral pitting edema was assessed by applying a gentle pressure with the thumb for 3 to 5 s to the dorsal surface of both feet and graded on a scale from 1 to 3, corresponding to edema detected on feet, feet and legs or including hands and face. The degree of edema at admission was reclassified as either mild (grade 1 and 2) or generalized edema (grade 3). Vital signs (axillary temperature, respiratory rate and pulse rate) were recorded by trained study nurses at admission.

As timing of follow-up measurements was based on the clinical improvement of the individual child, the time span between measurements varied. Days spent in the hospital were calculated for each child as well as days spent in each of the phases.

Information on co-diagnoses was extracted from the child’s clinical record.

### Blood collection and laboratory analysis

Venous blood was collected in dry tubes (SST™ Tubes, Becton Dickinson A/S) for separation of serum. Serum was separated within an hour after collection and kept at −80 °C for later analysis. Phosphate, magnesium and albumin were measured in serum using the automated Architect C4000 system (Abbott Diagnostics, USA). S-phosphate was determined by the phosphomolybdate method (Ref. no. 7D71, Abbott). The reference range, provided by the laboratory, for children 0–2 years was 1.45–2.10 mmol/L; and for 2–12 years: 1.45–1.78 mmol/L. S-magnesium was measured by the xylidyl blue method (Ref. no. MG531, Randox, UK). Interference from calcium was prevented by incorporation of a calcium chelating agent. The reference range, as provided by the laboratory, for children 5 months to 6 years was 0.70–0.95 mmol/L. Serum albumin (S-albumin) was measured by the bromcresol green method (ref no: 7D53-20, Abbott). The reference range for children (4 days–14 years) was 38–54 g/L according to the manufacturer.

The acute-phase protein alpha_1_-acid glycoprotein (AGP) was measured in serum using the automated Humastar 80 analyser (Human Diagnostics, Wiesbaden, Germany) and analyzed using polyclonal rabbit anti-human AGP antibody (code Q0326, DAKO Denmark A/S, Glostrup, Denmark).

Potassium was measured in serum, but not reported here as measurements are very sensitive to hemolysis. Hemolysis was present to some degree (visual inspection) in most samples and in particular in baseline samples, most likely reflecting the difficulties in collecting blood at this severe stage.

### Treatment

Treatment was based on the Ethiopian guideline for the management of children with SAM [20] and consisted of a stabilization, transition and rehabilitation phase. Therapeutic diets used in the NRU were the pre-mixed F-75 and F-100 (Nutriset, Malaunay, France), containing 560 and 579 mg/L of phosphorus and 85 and 154 mg/L of magnesium, respectively when mixed with water as per manufacturer specifications. At admission the children were given F-75 (100 kcal/kg/day). When appetite returned and/or edema had started to resolve, F-75 was replaced with an equal volume of F-100 (130 kcal/kg/day). This marked the start of the transition phase. Once the appetite was good and detectable edema had resolved the child was moved to the rehabilitation phase where F-100 was given at full dose (200 kcal/kg/day).

During the first 3 months of 2012 there was a national shortage of F-75 and all children admitted were stabilized with diluted F-100 in place of F-75. F-100 was diluted to an energy density of 75 kcal/100 mL, by adding 2700 mL instead of 2000 mL of water to one sachet of F-100 (456 g).

### Ethics

The study was approved by the College of Public Health and Medical Science ethical review board, Jimma University (Ref. no. RPGC/229/2011 and RPGC/16/2012). A consultative approval was obtained from the Danish National Board of Research Ethics. All parents/guardians gave oral and written consent to participate, after receiving oral and written information about the study and examinations, in local language. Parents/guardians were informed that they could withdraw from the study at any time without compromising the treatment of their child. Study blood samples were analyzed after completion of the study and results were not available to clinicians, and thus could not affect the treatment given. All study participants received standard treatment for their nutritional and additional co-morbidities according to the national protocol and the hospital treatment guide, respectively.

### Data handling and statistical analysis

Data was double entered using EpiData (version 3) and analysed using Stata 12 (Stata/IC) (StataCorp LP, College station, Texas, USA)).

Analysis of variance (ANOVA) was used to test for differences in means and chi-square test to test for differences in proportions between groups (i.e. children with and without edema; children stabilized using F-75 and diluted F-100). Data on days spent in each phase were transformed before analysis. As some children were directly transferred from stabilization to rehabilitation phase (zero values) data were further transformed prior to analysis by “*log (1+ days in transition phase)”*.

Analysis of covariance was used to identify predictors of baseline phosphate and magnesium while adjusting for age and sex.

Change over time in S-phosphate and S-magnesium, with adjustment for age, sex and edema, were analyzed using multilevel linear mixed-effects regression model with child-specific random effects, as repeated measurements per child were included in the analysis. Fixed effects included diet fed during stabilization phase (i.e. F-75 or diluted F-100) and treatment phase. Models were checked by visual inspection of standardized residual plots. Model estimates of mean (95 % CI) S-phosphate and S-magnesium are presented in the figures. Post hoc comparison of the effects between children stabilized using F-75 and diluted F-100 at different time points was performed using linear combinations of coefficients via lincom commands in STATA.

## Results

From May 2011 to April 2012 a total of 200 children aged 6–59 months were admitted with SAM; 37 children were not eligible due to critical illness (mainly respiratory difficulties) and of 163 eligible children, guardians of 72 (44 %) accepted the child’s participation in the study. Children included in the study were older than children not included (32 vs 26 months, *P* = 0.003), but there were no differences in sex distribution and proportion with edema.

Most children came from subsistence farming families outside Jimma town. Edema was present in 50 (69 %) of which 28 (56 %) had mild and 22 (44 %) generalized edema. Anthropometric, clinical and biochemistry data are presented in Table 1, separately for children with or without edema.

**Table 1:** Anthropometric, clinical and biochemistry data for 72 severely malnourished children at admission, by presence of edema^a^

	Edema	No edema	*P*
	(*n* = 50)	(*n* = 22)	
Female sex	24 (48.0)	8 (36.4)	0.36
Age, months	35 ± 14	26 ± 12	0.01
Anthropometric data			
Weight-for-height, z	−2.9 ± 1.6	−4.3 ± 0.9	<0.001
Height-for-age, z	−3.0 ± 2.0	−3.9 ± 1.8	0.059
Mid-upper arm circumference, cm	11.6 ± 1.5	9.5 ± 0.9	<0.001
Clinical data			
Axillary temperature, °C	36.3 ± 0.8	36.0 ± 0.6	0.11
Respiratory rate, breaths/min	32.1 ± 4.0	32.2 ± 3.3	0.91
Pulse rate, beats/min	113.4 ± 7.8	114.2 ± 6.6	0.68
Reported morbidity previous month			
Fever	40 (80.0)	17 (77.3)	0.79
Malaria	15 (30.0)	6 (27.3)	0.82
Cough or difficult breathing	15 (30.0)	11 (50.0)	0.10
Diarrhea	43 (86.0)	19 (86.4)	0.97
Measles	5 (10.0)	0 (0.0)	0.12
Morbidity ≥ 1	50 (100)	21 (95.5)	0.13
Co-diagnoses			
HIV	0 (0.0)	2 (9.1)	0.03
Tuberculosis	0 (0.0)	3 (13.6)	0.01
Persistent diarrhea	2 (4.0)	0 (0.0)	0.34
Pneumonia	13 (26.0)	7 (31.8)	0.61
Co-diagnoses ≥ 1	15 (30.0)	9 (40.9)	0.37
Biochemistry data			
Serum albumin, g/L	13.6 ± 6.4	24.1 ± 8.4	<0.001
Serum alpha_1_-acid glycoprotein, g/L	2.9 ± 0.8	2.7 ± 1.0	0.39

^a^ Values presented are mean ± SD or n (%). In 4 children no blood was obtained at admission. 12 blood samples were not analyzed for alpha_1_-acid glycoprotein

Twenty-five (35 %) of the children were admitted during a time period were there was a national shortage of F-75 and were stabilized with diluted F-100 in place of F-75. The 25 children fed diluted F-100 during stabilization did not differ from those fed F-75 (results not shown) in sex, age, proportion with edema, anthropometric measures and reported morbidity. There was, however a higher proportion diagnosed with pneumonia among those that received F-75 (38 vs 8 %, *P* = 0.006).

**Fig. 1:**
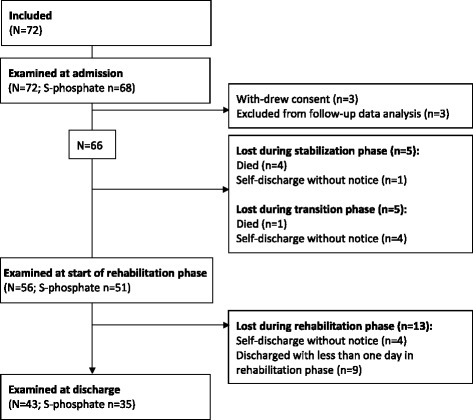
Flow diagram of the study participants

Of the 72 children enrolled, 6 were not followed up (3 withdrew consent, 3 received ready-to-use therapeutic foods). Of the remaining 66 children, 52 were followed until discharge, 8 until self-discharge and 5 until time of death (Fig. 1). Blood was collected and S-phosphate and S-magnesium determined in 68 (94 %) children at admission, 51 (91 %) at start of rehabilitation phase and 35 (81 %) at discharge.

The median (range) duration of stabilization, transition and rehabilitation phase was 6 (2–27), 2 (0–10) and 6 (1–22) days, respectively, and was not different between children with and without edema or in children fed diluted F-100 compared to those fed F-75 during stabilization (data not shown).

### S-phosphate and S-magnesium at admission

At admission mean ± SD S-phosphate was 0.92 ± 0.34 mmol/L and 63 (93 %) children had values below the age specific cut off defining normal values (<1.45 mmol/L). Mean ± SD S-magnesium was 0.95 ± 0.23 mmol/L, 9 (13 %) children had values below the age specific cut off defining normal values (<0.70 mmol/L) and 33 (49 %) children had values above (>0.95 mmol/L).

### Correlates of S-phosphate and S-magnesium at admission

Correlates of S-phosphate and S-magnesium are presented in Table 2. At admission S-phosphate was positively associated with S-magnesium (data not shown) and both were strongly positively correlated with S-albumin (0.02, 95 % CI: 0.01; 0.03 mmol/L, *P* < 0.001 and 0.02, 95 % CI: 0.01; 0.02 mmol/L, *P* < 0.001, respectively). The mean differences in S-phosphate (−0.13, 95 % CI: −0.31; 0.05 mmol/L) and S-magnesium (−0.12, 95 % CI: −0.24; 0.01 mmol/L) were not significant between edematous and non-edematous children.

**Table 2:** Correlates of serum phosphate and magnesium at admission in 72 children with severe acute malnutrition^a^

	[N]	S-phosphate, mmol/L		S-magnesium, mmol/L	
		B (95 % CI)	*P*	B (95 % CI)	*P*
Female sex	[68]	0.10 (−0.06; 0.27)	0.21	0.03 (−0.08; 0.14)	0.63
Age, months	[68]				
6–24	[29]	−0.15 (−0.34; 0.03)	0.10	−0.03 (−0.16; 0.10)	0.63
25–36	[22]	ref.		ref.	
37–60	[17]	−0.27 (−0.48; −0.06)	0.01	−0.14 (−0.28; 0.01)	0.07
Edema	[68]	−0.13 (−0.31; 0.05)	0.16	−0.12 (−0.24; 0.01)	0.07
Anthropometric data					
Mid-upper arm circumference, cm	[58]	0.01 (−0.04; 0.06)	0.74	−0.01 (−0.05; 0.02)	0.51
Weight-for-height, z	[67]	−0.02 (−0.07; 0.04)	0.51	−0.02 (−0.06; 0.02)	0.27
Height-for-age, z	[68]	−0.02 (−0.06; 0.02)	0.37	−0.02 (−0.05; 0.01)	0.12
Serum biochemistry:					
Serum alpha-1-Acid glucoprotein, g/L	[55]	−0.01 (−0.11; 0.10)	0.92	−0.02 (−0.09; 0.05)	0.54
Serum albumin, g/L	[68]	0.02 (0.01; 0.03)	<0.001	0.02 (0.01; 0.02)	<0.001
Co-diagnoses:					
Pneumonia	[68]	−0.07 (−0.25; 0.11)	0.46	−0.03 (−0.16; 0.09)	0.60
Tuberculosis	[68]	−0.10 (−0.58; 0.37)	0.66	0.20 (−0.12; 0.53)	0.22
One or more co-diagnoses ^b^	[68]	−0.05 (−0.22; 0.12)	0.58	−0.01 (−0.13; 0.11)	0.85

^a^ Data are regression coefficients B and 95 % confidence intervals adjusted for age and sex

^b^ Co-diagnoses included HIV, tuberculosis, persistent diarrhea and pneumonia

### S-phosphate and S-magnesium concentrations at admission by stabilization diet

Age- and sex-adjusted mean S-phosphate at start of treatment was higher in children admitted during the national shortage of F-75 (i.e. fed diluted F-100 during stabilization) compared to those fed F-75 (1.13 vs 0.95 mmol/L, *P* = 0.03) as was adjusted mean S-magnesium (1.08 vs 0.94 mmol/L, *P* = 0.01). The differences in S-phosphate and S-magnesium persisted after adjustment for pneumonia (data not shown).

#### Changes during treatment

**Fig. 2:**
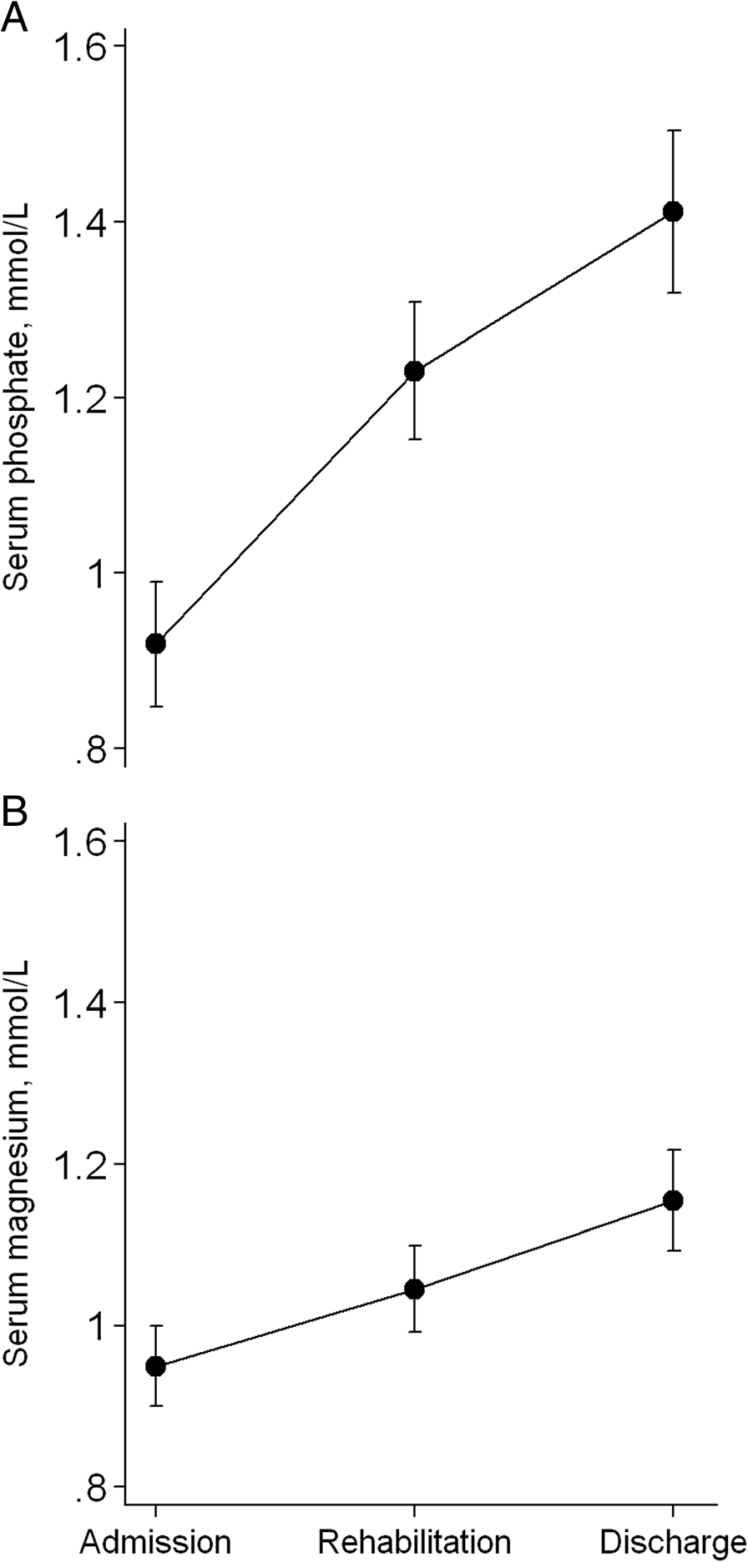
Estimated mean serum phosphate (**a**) and serum magnesium (**b**) concentrations during treatment in 66 children with severe acute malnutrition, shown with 95 % confidence intervals

**Fig. 3:**
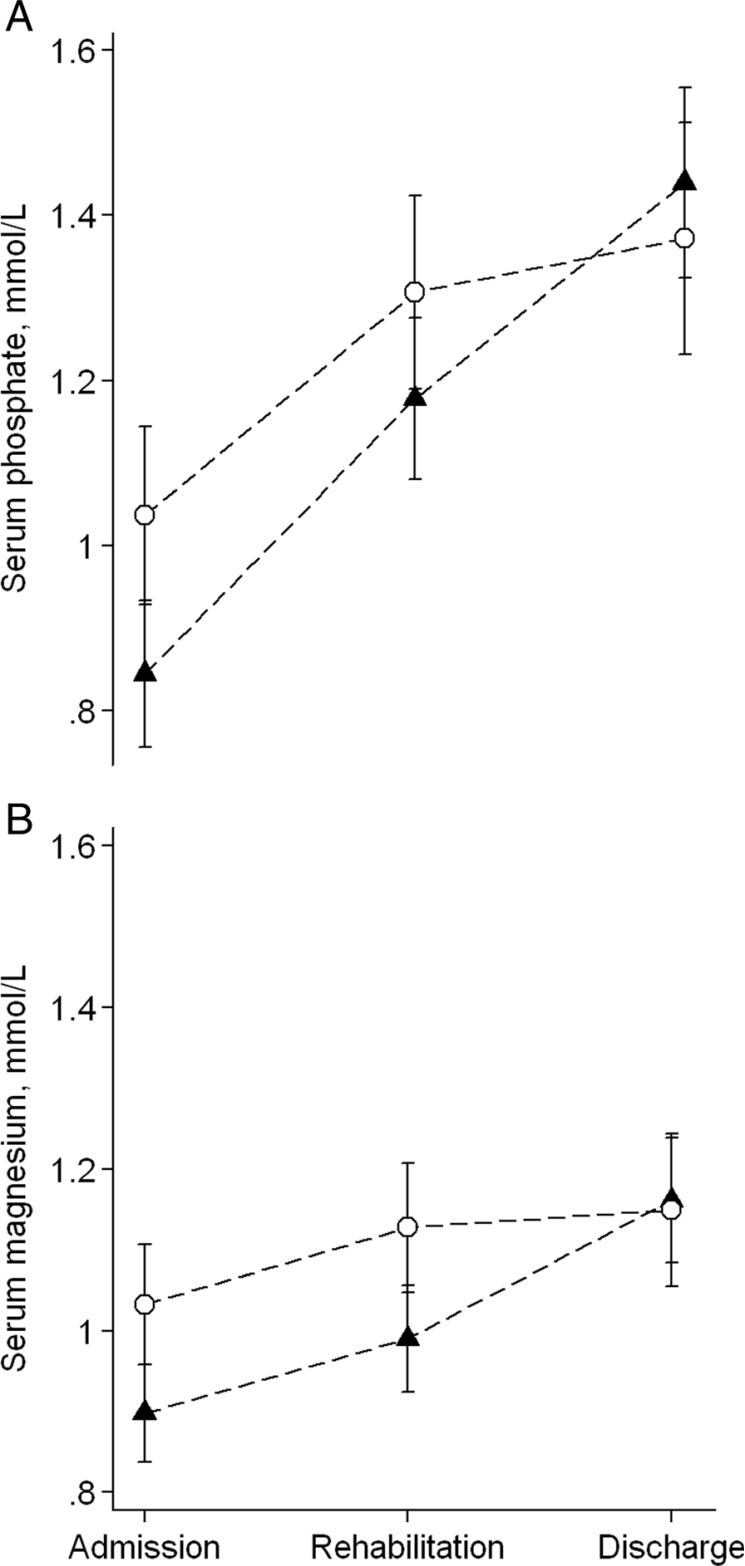
Estimated mean serum phosphate (**a**) and serum magnesium (**b**) concentrations during treatment, by diet fed during stabilization phase (F-75 (triangle, *n* = 41); diluted F-100 (circle, *n* = 25)) shown with 95 % confidence intervals, adjusted for sex and age and edema

Mean S-phosphate increased by 0.49 (95 % CI: 0.39; 0.60) mmol/L and S-magnesium by 0.21 (95 % CI: 0.14; 0.27) mmol/L during treatment (Fig. 2). At start of rehabilitation phase, S-phosphate in children stabilized with diluted F-100 was not different from children fed F-75 (Estimated mean difference: 0.13 (95 % CI: −0.02; 0.28) mmol/L, *P* = 0.099). There was no difference in S-phosphate at discharge between children receiving F-75 and diluted F-100 (*P* = 0.47). The higher S-magnesium observed at admission in children stabilized with diluted F-100 persisted at the start of the rehabilitation phase (estimated mean difference: 0.14 (95 % CI: 0.03; 0.24) mmol/L, *P* = 0.01) but at discharge S- magnesium was not different between children initially stabilized on F-75 and diluted F-100 (Fig. 3). The *p*-values for diet-time interactions were 0.046 and 0.056 for S-phosphate and S-magnesium, respectively.

### S-phosphate and S-magnesium concentrations at discharge

At discharge, 18 (51 %) children had S-phosphate below normal range for children, and 3 (9 %) had S-phosphate above. Most of the children (29 (83 %)) had S-magnesium above normal range for children.

## Discussion

We found that S-phosphate was low at admission among children with SAM, consistent with what others have reported [1, 2, 5, 21, 22]. S-magnesium was above normal range in the majority of the children at admission, although there was a large variation which is in accordance with previous reports [23]. Some studies have reported normal S-magnesium in the presence of magnesium deficits (indicated by low levels of magnesium in muscle tissue and low urinary excretion of magnesium) in children with SAM [24, 25], whereas one study found low S-magnesium in severely malnourished children with history of diarrhoea [3]. S-magnesium could be falsely increased due to mild haemolysis of blood samples; however, the degree of haemolysis was highest in blood samples collected at admission, where magnesium levels were lowest.

Both S-phosphate and S-magnesium correlated positively with S-albumin. The correlation of S-phosphate and S-magnesium with S-albumin is in accordance with some studies [26, 27], but in contrast to other studies [1, 2]. The correlation might be explained by the fact that phosphate and magnesium in serum are partially bound to albumin, or because the same factors causing low albumin in children with SAM also cause low S-phosphate and S-magnesium.

### Changes during treatment

We observed a steady increase in S-phosphate and S-magnesium during treatment, both among children with edematous and non-edematous malnutrition. For S-phosphate, however, 51 % of children still had levels below normal on discharge. This might be problematic for further catch up, as local diets are low in bioavailable phosphorus. The large number of children lost to follow-up, however, did not allow us to further explore predictors of S-phosphate at discharge.

The overall increase in S-phosphate and S-magnesium are reassuring; however, it is still unknown whether a higher content of particularly phosphorus would result in a faster normalization of S-phosphate, and whether this would improve outcome in terms of growth or mortality. While many children at discharge still have subnormal serum concentrations of phosphate, magnesium concentrations seem to be successfully corrected in most children, and perhaps even over-corrected. The importance of supplementing malnourished children with magnesium has been appreciated for many years, due to its importance to lean tissue accretion, as well as for homeostasis of K, Ca and other nutrients, and therefore F-75 and F-100 are fortified with high levels of Mg [17]. However, it is unknown whether the high concentration observed represents a true surplus of magnesium in the diet. The finding does however not support that the level of magnesium in F-100 should be the limiting nutrient for growth, as suggested [17].

Ideally, evaluation of the adequacy of current magnesium and phosphorus content of F-75 and F-100 would require a trial of nutritional products with different levels, and with measurement of urinary excretion and growth. It would also have been desirable to have measured S-phosphate on day two, to assess whether the currently used diets are able to prevent the previously described *phosphate nadir* after starting refeeding.

### Feeding diluted F-100 compared to F-75

Ideally, blood samples should have been taken before the first feed was given, but in practice this was almost impossible as initiation of treatment had highest priority and routine management does not include blood sampling. All blood samples were taken within the first 24 h after, but after the first feed was given. It is thus possible that the higher admission levels of S-phosphate and S-magnesium in children stabilized using F-100 could be caused by the diet. In support of this, previous studies have found that changes in S-phosphate during the first days of treatment depend on the type of diet given and that a *phosphate nadir* may occur during the first couple of days of treatment [1, 2]. The higher S-phosphate concentration at admission in children fed diluted F-100 is however somewhat surprising, as diluted F-100 contains less phosphorus than F-75. Acute infections have been associated with hypophosphatemia [28], but we did not find that the higher proportion of pneumonia among children fed F-75 could explain the lower S-phosphate. Another possible explanation could be that diluted F-100 provides less energy derived from carbohydrate and more from fat, possibly blunting the insulin response to refeeding, causing less flux of phosphate into the cells. Furthermore, the main source of phosphorus in F-100 is milk, which may be better absorbed than the inorganic phosphate salts with which F-75 is fortified. The study was not randomized to assess the effect of diluted F-100, and therefore our results should be interpreted with caution, and ideally re-tested in other settings.

### Strengths and limitations

This is one of the first studies to describe the pattern of P-phosphate in children with SAM receiving standard pre-mixed F-75 and F-100 fortified with phosphorus. A number of limitations pertain to the presented data. First, the observational design precludes conclusions about causality. Second, with the modest sample size, the power to detect differences was limited. Furthermore, it proved very difficult to get frequent blood samples in the first few days, as originally planned, so the second blood sample was postponed until the end of transition phase where the children were stable. Furthermore, it would have been ideal to take the first blood sample before the first feed, to be able to describe the acute effect of initiation of treatment. Finally, blood sampling was not complete and loss to follow-up was considerable which could introduce selection bias.

## Conclusion

Hypophosphatemia was common among children with SAM at admission, and still subnormal in about half of the children at discharge. This could be problematic for further recovery as phosphorus is needed for catch-up growth and local diets are likely to be low in bioavailable phosphorus. The high S-magnesium levels at discharge does not support that magnesium should be the limiting nutrient for growth in the F-100 diet. Although diluted F-100 (75 kcal/100 mL) is not designed for stabilizing children with SAM, it did not seem to cause lower S-phosphate than in children fed F-75.

## Abbreviations

AGP: Alpha_1_-acid glycoprotein
F-100: Therapeutic formula used in transition and rehabilitation phase of in-patient treatment for SAM
F-75: Therapeutic formula used during stabilization phase of in-patient treatment for SAM
JUSH: Jimma University Specialised Hospital
MUAC: Mid-upper arm circumference
NCHS: National Center for Health Statistics
NRU: Nutritional rehabilitation unit
S-albumin: Serum albumin
SAM: Severe acute malnutrition
S-magnesium: Serum magnesium
S-phosphate: Serum phosphate
WHO: World Health Organization
